# Hot Mitochondria and the Second Law of Thermodynamics

**DOI:** 10.21203/rs.3.rs-8744427/v1

**Published:** 2026-02-20

**Authors:** Alexei Tkachenko, Belem Yoval-Sánchez, Alexander Galkin

**Affiliations:** 1 Center for Functional Nanomaterials, Brookhaven National Laboratory, Upton, NY, USA; 2 Feil Family Brain and Mind Research Institute, Weill Cornell Medicine, New York, NY, USA

## Abstract

Mitochondria are central hubs of cellular bioenergetics, converting chemical free energy into ATP while inevitably releasing heat during respiration. Fluorescence-based thermometry has been interpreted to show intracellular “hot spots” more than 10 °C above the bulk physiological temperature, implying that mitochondria might operate far outside conventional thermal bounds. Such claims, however, appear inconsistent with basic biophysics: the small size of mitochondria, their aqueous and highly conductive environment, and their limited power output all argue against large steady-state temperature gradients. This discrepancy has prompted renewed scrutiny of both the physical limits of intracellular heat transfer and the biological interpretation of nanoscale thermal measurements. A key open question is whether nonequilibrium biochemical processes, such as respiration-driven proton pumping, could act as nanoscale heat pumps that maintain higher local temperatures than allowed by passive diffusion alone. Here, we develop a model-independent thermodynamic analysis based solely on the Second Law of Thermodynamics to bound the maximal temperature difference that any biochemically driven mechanism can sustain across the inner mitochondrial membrane and show that even under idealized conditions the achievable temperature rise is restricted to a small fraction of a degree, effectively closing this loophole.

## I. INTRODUCTION

**FIG. 1.**
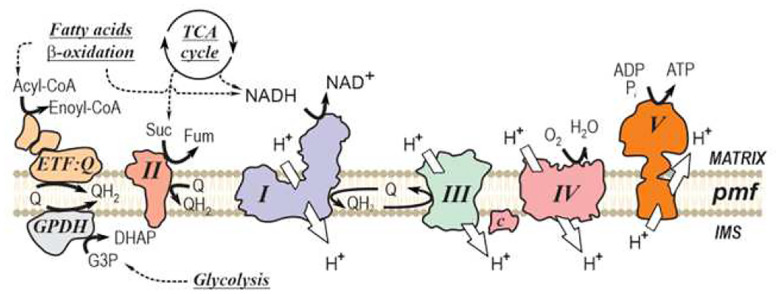
Oxidative phosphorylation and major electron entry points to the respiratory chain. Schematic of the inner mitochondrial membrane showing the respiratory chain (Complexes I–V) and other dehydrogenases that feed electrons to the ubiquinone Q/QH_2_ pool. Electrons from NADH (generated by the TCA cycle, oxidation of fatty acids, or the malate–aspartate shuttle) enter via Complex I, while succinate is oxidized to fumarate by Complex II. Additional inputs arise from mitochondrial glycerol-3-phosphate dehydrogenase (GPDH), which oxidizes glycerol-3-phosphate (G3P) to dihydroxyacetone phosphate (DHAP), and from fatty-acid β-oxidation through the electron-transferring flavo-protein:ubiquinone oxidoreductase (ETF:Q), which accepts electrons from acyl-CoA dehydrogenases (Acyl-CoA → Enoyl-CoA). Reduced ubiquinone (QH_2_) delivers electrons to Complex III, which passes them via cytochrome c to Complex IV, where O_2_ is reduced to H_2_O. Proton translocation by Complexes I, III, and IV (white arrows) from the matrix to the intermembrane space (IMS) establishes the proton-motive force (pmf) across the inner membrane, which is used by ATP synthase (Complex V) to drive ATP formation. Solid black arrows indicate electron flow; dashed arrows indicate metabolic pathways and shuttles that supply reducing equivalents.

Cellular aerobic catabolism relies on the breakdown of sugars, lipids, and proteins into smaller molecules, followed by their oxidation in mitochondria through a series of exergonic reactions. A fraction of the released free energy is conserved and stored in the form of adenosine triphosphate (ATP), the universal energy currency of all cellular life forms. This process was termed oxidative phosphorylation by W. Engelhardt in the late 1930s [1, 2], with its components located at the inner mitochondrial membrane (Fig. 1). During the oxidative phase, several respiratory substrates (NADH, succinate, glycerol 3-phosphate, acyl-coenzyme A, and others) are oxidized by membrane-bound dehydrogenases. The derived electrons are transferred to the membrane-soluble carrier ubiquinone and subsequently passed to Complex III. From there, electrons are shuttled via cytochrome c to Complex IV, where molecular oxygen serves as the terminal electron acceptor, producing water. The free energy released during these redox reactions drives proton translocation by Complexes I (NADH dehydrogenase), III, and IV across the inner membrane, from the matrix to the intermembrane space, generating the proton-motive force (pmf) (Fig. 1). During the phosphorylation phase, the backflow of protons from the intermembrane space to the matrix drives the synthesis of ATP from ADP and inorganic phosphate at Complex V or ATP synthase. In aerobic cells, the majority of ATP is produced in mitochondria via oxidative phosphorylation.

The thermodynamic characteristics of ATP production during oxidative phosphorylation have been examined by thousands of researchers over the past century. The thermodynamic efficiency of the ATP formation process, i.e., the percentage of the total free energy change that is stored in the form of ATP, is often regarded as being high (~ 60–70%) [3]. When the rates of substrate oxidation and ATP synthesis are taken into account, it has been estimated that mitochondria dissipate about 80% of the energy derived from oxidized substrates as heat [4]. This observation suggests that, under certain conditions, the released heat could potentially increase the local temperature in the vicinity of mitochondria, with some reports proposing values as high as 10–15 °C above the surrounding environment [5]. Most estimations assume that mitochondria are membrane-formed spheres, with heat production occurring across the surface [6, 7]. However, *in situ*, heat is produced across the cristae membranes arranged in parallel, which may help to transiently retain heat within the mitochondrial matrix [8].

Several experimental approaches have been developed to assess mitochondrial heat production and temperature changes in intact cells [9]. These include mitochondria-targeted fluorescent proteins such as tsGFP1-mito, gTEMP, emGFP-Mito [10–12], fluorescent polymers [13], and low-molecular-weight sensors like rhodamine, Mito-RTP [14–17], rhosamine-MTY [5], or BODIPY-based reporters [18]. Application of these probes has revealed temperature heterogeneity within a cell. However, a number of biochemical factors such as pH, calcium concentration, membrane potential, and redox state can influence probe fluorescence, potentially leading to apparent signal changes that may be misinterpreted as temperature fluctuations [5, 19]. Therefore, the conclusions of Chrétien *et al*. [5] were challenged by direct measurements of the effects of temperature on isolated intact mouse liver mitochondria. These studies demonstrated that the stability of respiratory complexes and supercomplexes declines sharply at temperatures above 43 °C, indicating that such high temperatures cause structural and functional damage to mitochondrial machinery. At the same time, mitochondrial-respiration-mediated temperature increase contradicts established principles of physics governing energy transfer, including absolute heat release, thermal conductivity, and the geometric constraints of the cellular environment.

In this work, we re-examine the physical limits of mitochondrial temperature using two complementary approaches. First, we refine the classical diffusion-based estimate, incorporating realistic mitochondrial geometry and maximal metabolic power rather than relying on a coarse cell-level approach. This analysis confirms that even under extreme uncoupled respiration, the steady-state surface temperature rise remains extremely small. Second, we address the plausible loophole in diffusion-based arguments: the possibility that nonequilibrium biochemical activity could actively sustain internal temperature gradients. To examine this scenario, we derive a general bound, based on the Second Law of Thermodynamics, which establishes that “the entropy of a closed system tends to increase over time,” on the largest temperature difference that any biochemically powered heat-pump mechanism could maintain across the inner membrane. By combining this thermodynamic constraint with the known geometric and biophysical parameters, we demonstrate that even the most favorable nonequilibrium mechanisms cannot produce more than a fraction of a degree of heating.

## II. RESULTS

### A. Thermal diffusion result: the 10^5^ gap

We begin by refining the standard estimate of the temperature difference between a mitochondrion and its surroundings, based on Fourier’s law of thermal diffusion:

(1) J=-κ∇T,

where J is the heat flux (power transfer per unit area), ∇T is the temperature gradient, and κ is the thermal conductivity inside the cell. A natural reference value for κ is that of water, $\kappa \approx 0.6\;\text{W}{\;\text{m}}^{-1}{\;\text{K}}^{-1}$ [20], although recent reports suggest that the cytoplasmic conductivity may be significantly lower, $\kappa \approx 0.1\;{\text{W m}}^{-1}{\;\text{K}}^{-1}$ [21].

Mitochondria can exist as interconnected tubular networks with a characteristic radius $r_{0}\approx 0.2-0.5\;\mu \text{m}$, or as fragmented spheroids connected end-to-end [22]. Applying Fourier’s law at a distance r from the mitochondrial surface gives

(2) $$\frac{dT}{dr}=-\frac{J(r)}{\kappa }=-\frac{J_{0}}{\kappa }\left\{\begin{array}{ll} {\left(\frac{r_{0}}{r}\right)}^{2}, & \text{spheroid,}\\ \frac{r_{0}}{r}, & \text{tubular.} \end{array}\right.$$

Here $J_{0}$ is the local heat flux in the vicinity of the mitochondrial surface. In our estimations we deliberately adopt the upper theoretical limit, assuming that *all* the energy released by mitochondrial respiration is converted to heat. Under such conditions, uncoupling agents render the inner membrane permeable to protons, collapsing the proton-motive force. While this accelerates respiration, ATP synthesis ceases, and 100% of the energy released from redox reactions is dissipated as heat. Maximum heat production by mitochondria is limited by their maximal respiratory capacity, defined by the respiratory complexes content [8].

(3) $$J_{0}=\frac{P}{A},$$

where P is the total mitochondrial power consumption (using 480 kJ/mol of O_2_ as the available free energy) and A is the surface area of the inner mitochondrial membrane.

In both geometries, spherical or tubular, to obtain dT/dr we consider the heat flux through the surface at distance r from the mitochondrion. The areas are $A(r)=4\pi r^{2}$ for a sphere and A(r)=2πrL for a cylinder of length L. Integrating the stationary heat equation over r yields the excess temperature at the mitochondrial surface:

(4) $$\Delta T=-\int_{r_{0}}^{r^{*}}\frac{dT}{dr}dr=\frac{r_{0}J_{0}}{\kappa }\left\{\begin{array}{ll} 1, & \text{spheroid,}\\ \frac{1}{2}\text{ln}\left(\frac{1}{\varphi }\right), & \text{tubular,} \end{array}\right.$$

where $r^{*}$ is the radius of the “neighborhood” surrounding the tubular mitochondrion, determined by the proximity criterion. By construction,

(5) $$\varphi =\frac{r^{*2}}{r_{0}^{2}}$$

is the volume fraction occupied by the mitochondrial network.

To estimate the power dissipation per unit area, we consider cardiomyocytes as the most metabolically intensive cell type [22]. It has been reported that they consume oxygen at rates as high as 1–2 fmol cell^−1^ s^−1^ [23, 24]. Taking an average cell volume of 35 pL [22], we estimate the mitochondrial heat production per cell volume as

(6) $$\frac{P}{V_{\text{cell}}}\approx \frac{480\;\text{kJ}/\text{mol}\times 2\;\text{fmol}/\text{s}}{35\;\text{pL}}\approx 30\;\text{W}/\text{L}.$$

The total mitochondrial volume of cardiomyocytes can reach ~ 40% of the cell volume [25, 26]. For tubular mitochondrial geometry, this volume can be related to surface area through

$$V_{\text{mito}}\approx 0.4\;V_{\text{cell}}\approx \frac{r_{0}A}{2}.$$

Putting these estimates together,

(7) $$J_{0}=\frac{P}{A}\approx 0.8\frac{r_{0}P}{V_{\text{cell}}}\approx 0.01\;{\text{W m}}^{-2}.$$

This value is consistent with alternative estimates [27], which set the typical power per mitochondrion in the range 0.1–0.01 pW. For a single mitochondrion with surface area $A\approx 1\;\mu {\text{m}}^{2}$, this power corresponds to a heat flux $J_{0}\approx 0.1-0.01\;{\text{W m}}^{-2}$. Some species and cell types may reach as high as $J_{0}\approx 1\;{\text{W m}}^{-2}$.

To estimate the upper bound for the temperature difference between the mitochondrial surface and its environment, we substitute

$$J_{0}\approx 1{\;\text{W m}}^{-2},\;r_{0}\approx 0.5\;\mu \text{m},\;\kappa \approx 0.1\;{\text{W m}}^{-1}{\;\text{K}}^{-1},\;\varphi \approx 0.1$$

into Eq. (4):

(8) $$\Delta T\approx \frac{J_{0}r_{0}}{2\kappa }\text{ln}\left(\frac{1}{\varphi }\right)≲10^{-5}\;{}^{\circ}\text{C}.$$

This reproduces the earlier estimates commonly referred to as the “10^5^ gap” [6, 7]. Note that our treatment explicitly focuses on geometry and heat production at the level of individual mitochondria, rather than using a coarse-grained cell-scale estimate. Taken alone, this refinement would predict an even smaller ΔT, but this effect is offset by our use of deliberately extreme parameter values, particularly for $J_{0}$ and κ.

### B. Thermodynamic bound on cross-membrane temperature gradient

At first sight, the above analysis appears robust and sufficiently general to rule out the idea of “hot” mitochondria. However, a loophole remains: mitochondria operate under strongly nonequilibrium conditions, where biochemical processes could, in principle, sustain internal temperature gradients by harnessing the available power, much like a standard heat pump that uses mechanical work to reverse the direction of heat flow [28]. Indeed, the inner mitochondrial membrane is an intricate nonequilibrium nanomachine, and from a fundamental standpoint it is conceivable that active reactions could function as a heat pump, generating a steady-state temperature difference between the matrix (interior) and the intermembrane space (exterior). For example, a recent model [29] suggests that ATP synthase could function as a microscopic “thermal ratchet,” producing a localized temperature gradient.

**FIG. 2.**
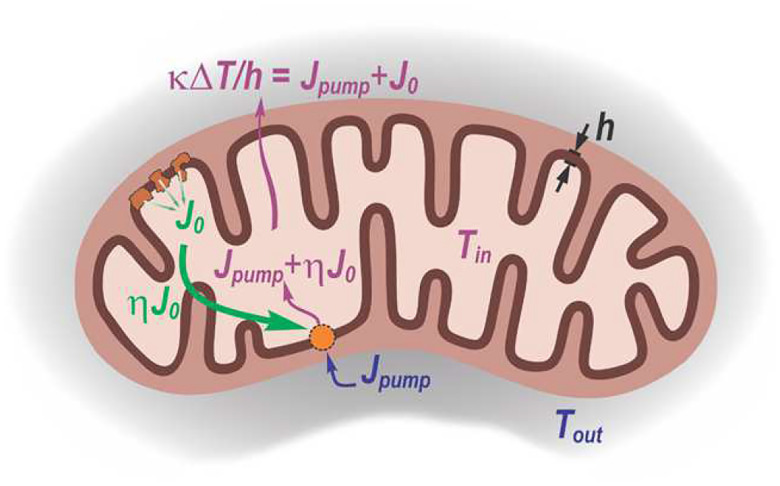
Mitochondrial inner membrane as a hypothetical heat pump. Schematic representation of plausible energy flows when the inner mitochondrial membrane (brown) operates as a biochemically powered heat pump. The inner membrane separates the mitochondrial matrix (pale yellow), at temperature $T_{\text{in}}$, from the surrounding medium (grey), at temperature $T_{\text{out}}$. Metabolic reactions by three proton-pumping respiratory chain complexes (dark orange) provide a power input $J_{0}$ (green), of which a fraction $\eta J_{0}$ is used to drive the heat pump. This pump transfers heat $J_{\text{pump}}$ from the exterior to the interior (blue), so that the total heat flux into the matrix is $J_{\text{pump}}+\eta J_{0}$ (magenta). This inward flux must be balanced by conductive heat loss across the membrane of thickness h, described by Eq. (9).

Figure 2 illustrates plausible energy flows when the inner mitochondrial membrane acts as a biochemically powered heat pump. The membrane separates the mitochondrial matrix (pale yellow) at temperature $T_{\text{in}}$ from the surrounding medium (grey) at temperature $T_{\text{out}}$. Metabolic reactions carried out by the three proton-pumping respiratory chain complexes (dark orange) provide a power input $J_{0}$ (green), of which a fraction $\eta J_{0}$ is used to drive the heat pump. This pump transfers heat $J_{\text{pump}}$ from the exterior to the interior (blue), so that the total heat flux into the matrix is $J_{\text{pump}}+\eta J_{0}$ (magenta). This inward flux must be balanced by conductive heat loss across the membrane of thickness h, described by

(9) $$\frac{\kappa \Delta T}{h}=J_{\text{pump}}+J_{0},$$

where κ is the thermal conductivity and $\Delta T=T_{\text{in}}-T_{\text{out}}$. The scheme illustrates the quantities used to derive the thermodynamic upper bound on the temperature difference across the inner membrane.

Below, we present a general, model-independent analysis that sets the upper bound on excess temperature inside mitochondria, based solely on the Second Law of Thermodynamics [28]. Specifically, we consider a scenario in which the inner mitochondrial membrane acts as a biochemically powered heat pump. In analogy to a classical heat engine, which uses a heat flow from a hotter to a colder reservoir to produce work, a heat pump operates in reverse, converting work to drive a heat flow from the colder to the hotter, thereby sustaining a temperature gradient (Fig. 2).

Let T be the temperature of the cellular environment, which we assume to be in equilibrium with the mitochondrial intermembrane space, and let $T_{\text{in}}>T$ be the temperature inside the inner membrane. As before, let $J_{0}$ denote the total metabolic power supplied per unit mitochondrial surface area. We assume the mitochondrion is not fully uncoupled, but instead may use a fraction $\eta J_{0}$ of this power to “pump” heat inward. Let $J_{\text{pump}}$ be the heat flux per unit area actively transferred from the exterior to the interior. The power $\eta J_{0}$ is expended to generate this inverse heat flow and returns as heat on the interior side. Thus, the total heat flux entering the matrix is

(10) $$J_{\text{in}}=J_{\text{pump}}+\eta J_{0}.$$

Applying the Second Law of Thermodynamics to this membrane heat pump gives the entropy production per unit area:

(11) $$\frac{dS}{dt}=\frac{J_{\text{pump}}+\eta J_{0}}{T_{\text{in}}}-\frac{J_{\text{pump}}}{T_{\text{out}}}\geq 0.$$

With $\Delta T=T_{\text{in}}-T_{\text{out}}$, inequality (11) implies

(12) $$J_{\text{pump}}\leq \eta J_{0}\frac{T_{\text{out}}}{\Delta T}.$$

A key property of a heat pump is that the transferred heat flux $J_{\text{pump}}$ can greatly exceed the mechanical or biochemical power consumption $\eta J_{0}$. We now analyze this extreme regime. Any heat pumped inward must be removed by ordinary thermal conduction across the inner membrane:

(13) $$J_{\text{pump}}=\kappa \frac{\Delta T}{h},$$

where κ is the membrane thermal conductivity and h its thickness.

Combining Eqs. (12) and (13) yields an upper bound for the cross-membrane temperature difference:

(14) $$\Delta T\leq \sqrt{\frac{\eta hJ_{0}T_{\text{out}}}{\kappa }}.$$

Most parameters in Eq. (14) are known with reasonable accuracy: $T_{\text{out}}\approx 310\;\text{K}$, membrane thickness h≈5nm. Based on experimental measurements of heat transport in lipid bilayers [30] and typical thermal conductivities of bulk fats [20], we adopt the lower-bound estimate $\kappa \approx 0.1\;\text{W}{\;\text{m}}^{-1}{\;\text{K}}^{-1}$ for the inner membrane. Using upper-bound values for the efficiency η≈1 and metabolic power per area $J_{0}\approx 1\;{\text{W m}}^{-2}$, we obtain

(15) $$\Delta T\leq 5\times 10^{-3}\;{}^{\circ}\text{C}.$$

This result significantly narrows the “10^5^ gap,” but still effectively rules out the possibility of a substantial temperature difference, even when nonequilibrium driving is taken into account. The new bound may appear to conflict with the ratchet model predicting multi-degree temperature differences [29]; however, that model assumes nearly perfect thermal isolation between compartments. Once the finite thermal conductance of the membrane is included, its predictions necessarily fall below the bound set by the Second Law.

## III. CONCLUSIONS

In this communication, we addressed the puzzling “10^5^ gap” between the reported mitochondrial “overheating” and the fundamental theoretical estimates based on thermal diffusion. After refining the diffusion-based analysis by incorporating realistic mitochondrial geometry and maximal metabolic power, we turned to a more challenging possibility: that mitochondria, as strongly nonequilibrium biochemical machines, might sustain internal temperature gradients beyond those allowed by passive heat diffusion [28, 29].

To address this, we derived a general thermodynamic bound on mitochondrial temperature gradients from the Second Law of Thermodynamics. Allowing the inner membrane to operate as an ideal, respiration-driven heat pump, we determined the maximal entropy-compatible inward heat flux. When this flux is balanced against unavoidable conductive losses, it yields a strict upper limit: even in the most favorable nonequilibrium regime, the temperature difference across the inner mitochondrial membrane cannot exceed a small fraction of a degree.

Our thermodynamic bound closes the loophole left by earlier diffusion-based analyses. We conclude that no molecular mechanism, regardless of complexity or efficiency, can generate or maintain multi-degree mitochondrial overheating without violating fundamental thermodynamic constraints. Consequently, large apparent “temperature” gradient signals reported by intracellular thermometer sensors are most likely due to biochemical modulation of their fluorescence rather than genuine thermal gradients.

## Data Availability

No datasets were generated or analyzed during the current study.
